# Case report: Diagnosis of NUT carcinoma of hepatic origin by next-generation sequencing

**DOI:** 10.3389/fonc.2023.1304457

**Published:** 2024-01-23

**Authors:** Bach Ardalan, Jose Azqueta, Jia Luo, Christopher French

**Affiliations:** ^1^ Sylvester Comprehensive Cancer Center, University of Miami Health System, Miami, FL, United States; ^2^ Department for Medical Oncology, Dana–Farber Cancer Institute, Boston, MA, United States; ^3^ Department of Pathology, Dana–Farber Cancer Institute, Boston, MA, United States

**Keywords:** NUT carcinoma, nextgeneration sequencing (NGS), hepatic tumor, NUT gene, rare representation

## Abstract

NUT carcinoma is a rare subcategory of squamous cell carcinoma. The latter is primarily characterized by the fusion of the coding sequence *NUTM1* on chromosome 15q14 with BRD4 or BRD3, both of which are acetyl-histone binding bromodomains. This tumor is often misdiagnosed due to its rarity and its histological similarity with other squamous cell carcinomas. It typically presents as a poorly differentiated squamous cell carcinoma in the head, neck, and mediastinal region, and has no distinct clinical characteristics that set it apart from other malignancies. Although uncommon, other NUT carcinomas have been reported in the literature outside of the midline region. Through next-generation sequencing, we were able to correctly diagnose our patient with the first-documented case of NUT carcinoma of hepatic-only origin.

## Introduction

Nuclear protein of testis (NUT) carcinoma is an uncommon and often highly aggressive malignancy of squamous cell carcinoma that is characterized by the fusion of the coding sequence *NUTM1* on chromosome 15q14 with BRD4 or BRD3, both acetyl-histone binding bromodomains. The diagnosis of these tumors is obtained via immunohistochemistry staining for expression of the *NUTM1* fusion protein (1). NUT carcinoma (NC) affects both sexes equally and can affect anyone regardless of age, however it tends to be diagnosed in the young. NUT carcinoma has a few reported cases every year. Due to lack of information on the subject, it is speculated that actual cases are much higher. Typically this tumor presents as a poorly differentiated squamous cell carcinoma originating in the head, neck, and mediastinal region; over the years, tumors arising outside the midline region have been documented.

This is the first reported case of NC of hepatic origin. This report describes the management of an adult female patient with a rare neoplasm originating from her liver, which required interdisciplinary collaboration and a review of the literature of NUT carcinomas arising from other organ sites.

## Case presentation

**Figure 1:**
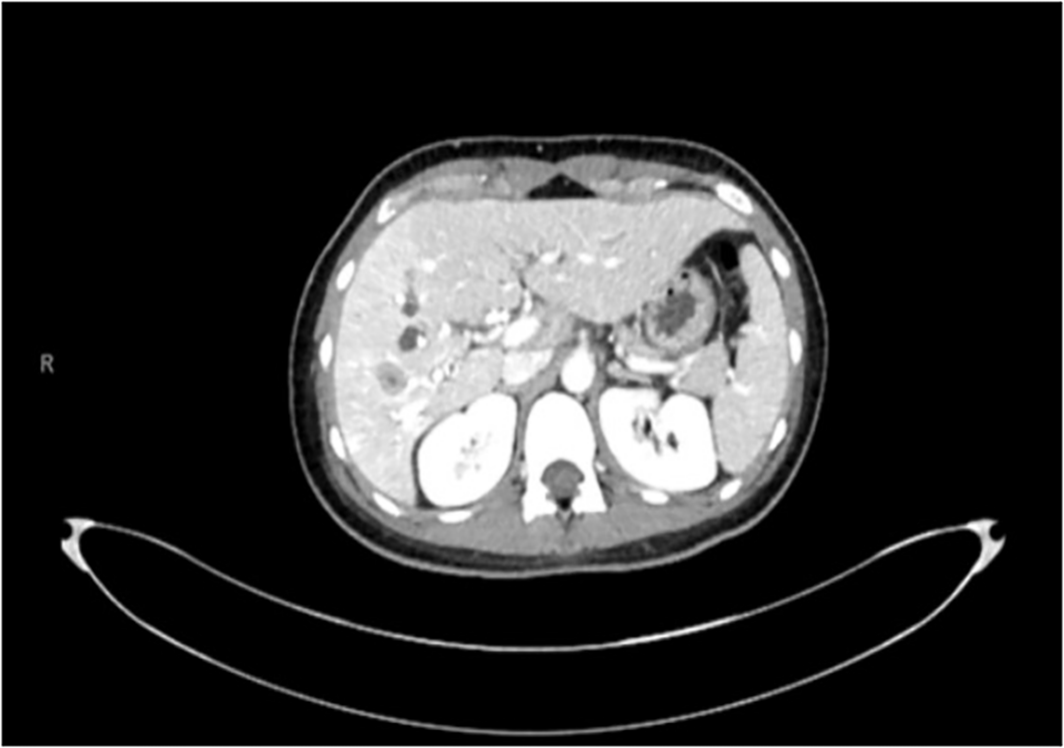
Initial CT scan dated July 2021 showcasing hypodense lesions on segment V of the liver.

An otherwise healthy female in 32-year-old with no familial history of cancer presented to her local emergency department complaining of worsening abdominal pain and obstructive jaundice in July 2021. Upon workup, she was found to have multiple masses on the right lobe of her liver (**Figure 1**). She underwent diagnostic laparoscopy and extended right hepatectomy with lymphadenectomy and hepaticojejunostomy. Surgical pathology was read as moderately differentiated cholangiocarcinoma with squamous differentiation, staged pT2bN1. Immunohistochemistry showed tumor cells expressed CK7, CK19 and were negative for synaptophysin, chromogranin, CD56 and arginase. She was placed on an adjuvant regimen of gemcitabine 1000 mg/m2 IV weekly and capecitabine 1000 mg orally twice daily. She soon developed a grade III maculopapular rash on her torso, thighs, and forearms which was attributed to gemcitabine by an allergist so ultimately only received 1 dose of gemcitabine and 5 days of capecitabine. Further treatment with IV chemotherapy was placed on hold. She felt that the rash was worsening after taking her evening pill of capecitabine. On the fifth day, she was advised to discontinue capecitabine. She was referred to and saw an allergist who found her to be allergic to gemcitabine.

**Figure 2:**
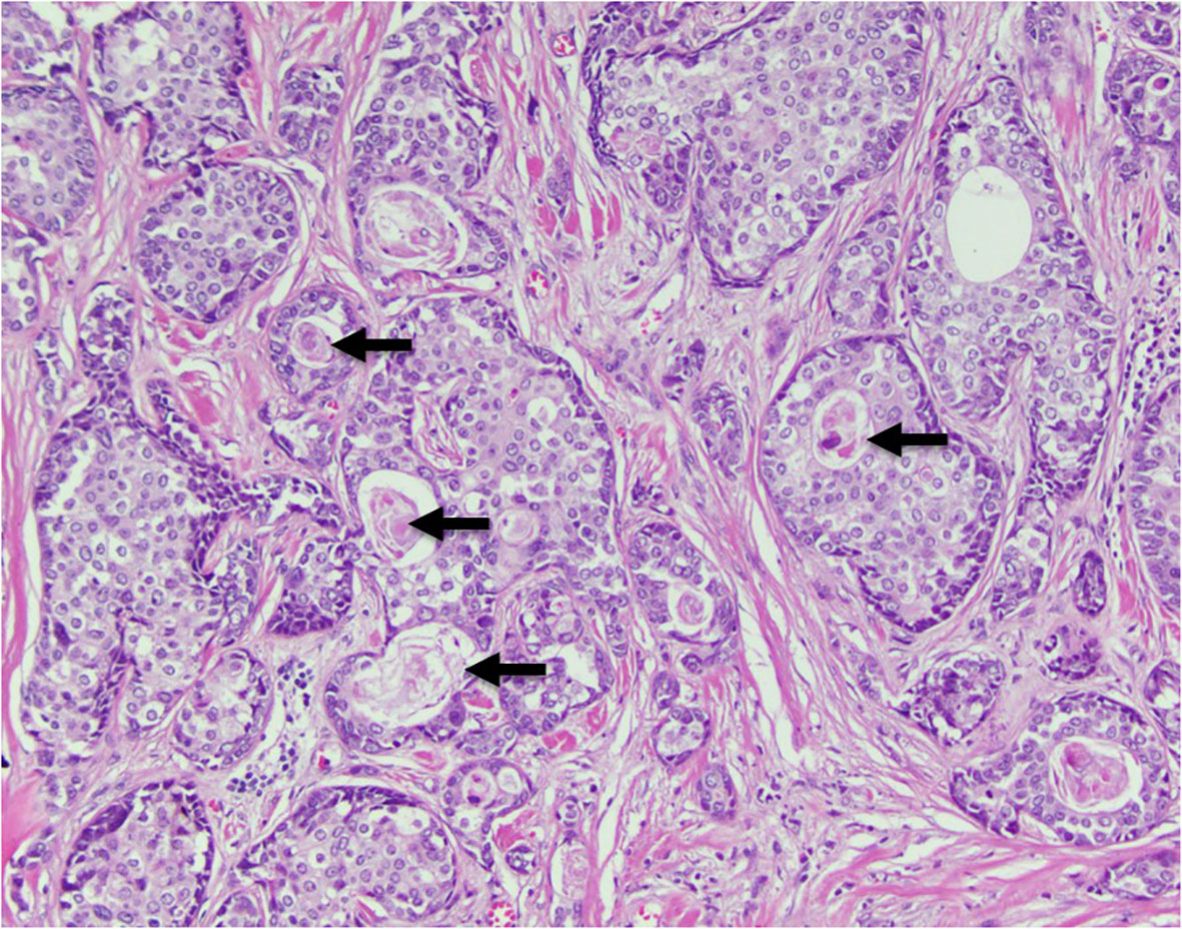
Histopathology section illustrating a nested growth pattern of relatively monotonous cells with moderate clear to eosinophilic cytoplasm and characteristic abrupt keratinization (arrows).

**Figure 3:**
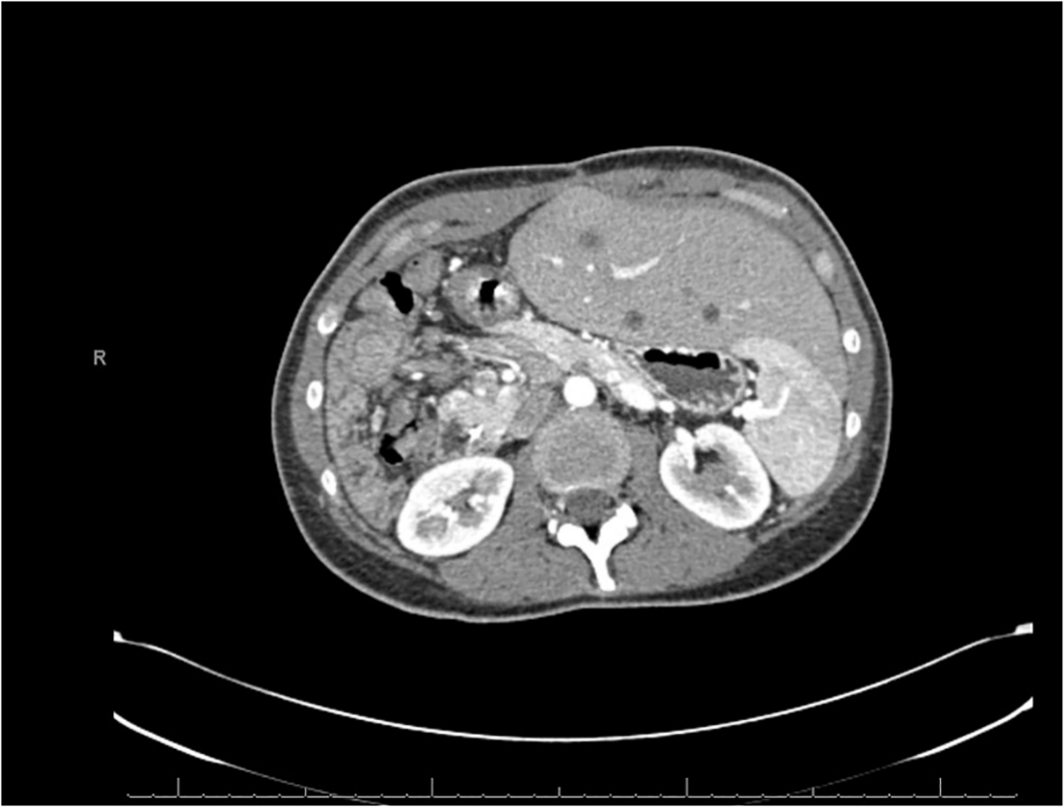
CT scan dated July 2022 showing recurrent lesions in the liver. The left lobe of the liver has grown significantly to compensate for the prior hepatic resection.

Five months later, she transferred her care to our institution and was seen in the outpatient clinic. There was discrepancy in the histopathologic diagnosis (**Figure 2**) by our pathology department. Her tumor sample was sent out to an external vendor for DNA and RNA-based next-generation sequencing. The external vendor provided a whole-genome analysis of the resected tumor. NGS was performed on genomic DNA isolated from the formalin-fixed paraffin-embedded (FFPE) tumor sample using the NextSeq or NovaSeq 6000 platforms, a pull-down panel designed to enrich over 700 clinically relevant genes was fused and another panel to enrich an additional 20,000 at lower depth. The report indicated a *BRD3*::*NUTM1* fusion via RNA sequencing; Exon 9 of *BRD3* was joined in-frame to exon 5 of *NUTM1*. Other results found on the final genomic report include: a tumor mutational burden of 4 mt/mb and Her2/Neu negative; no other relevant biomarkers noted. Prior to the initiation of therapy, CT and PET/CT scans were ordered to restage the patient. Imaging studies showed recurrent lesions in her remaining lobe of the liver and new lymphadenopathy; her left lobe of the liver had grown to compensate following prior partial hepatectomy (**Figure 3**).

She was placed on a modified FOLFOX regimen q2w and Pembrolizumab 200mg q3w and she received this medication over the course of 6 months. After 3 months, her scan showed stable disease. The patient tolerated the chemotherapy well with little to no secondary effects.

She was referred to be enrolled in an ongoing clinical trial (NCT05019716) involving combination therapy of cisplatin, etoposide, and BET inhibitor (ZEN003694).

## Discussion

NUT carcinoma, previously referred to as NUT midline carcinoma, is an uncommon and often misdiagnosed poorly differentiated carcinoma characterized by the translocation of the *NUTM1* gene (nuclear protein in testis)on chromosome 15q14. In the majority of cases *NUTM1* is fused to *BRD4* on chromosome 19p13.12. Moreover, the fusion of *NUTM1* to *BRD3* on chromosome 9q34.2 or *NSD3* on chromosome 8p11.23 may occur less frequently (2–5). Histologically NUT carcinomas tend to be poorly differentiated, often displaying squamous differentiation as seen in our patient (**Figure 2**). Its appearance is similar to other poorly differentiated and small round blue cell tumors, leading to frequent misdiagnosis of the tumor. Distribution of the NC has been observed equally in males and females, and in all age groups (6).

Presently, there are no standard of care therapies for treatment of NUT carcinoma. The majority of the patients have ultimately succumbed to the disease due to its aggressive nature, however there have been cases where up-front surgical resection of the tumor may improve OS (7). Several phase I trials have investigated the efficacy of direct inhibitors of the BRD4 portion of the BRD4-NUTM1 gene, dubbed “BET inhibitors” or BETi. These targeted inhibitors have been used as a monotherapy and have shown some efficacy despite toxicities (8).

It can be noted that the majority of cases reported arise from the midline region or mediastinum, however, there have been documented cases of NUT carcinoma originating outside of the midline region. Upon reviewing the literature, “non-midline” NC sites have been reported in the following tissues: thyroid, pancreas, bone, salivary gland, and soft tissues. A table of reported NC cases outside of the midline region seen in the literature is detailed on **Table 1** (1, 9–20). It is worth noting that the majority of fusion patterns in the literature were that of *BRD4-NUTM1*, whereas in our patient, her fusion was found to be *BRD3::NUMT1*. The significance of this is unknown, however, no other patient of non-midline origin have had the *BRD3::NUMT1 fusion*.

**Table 1:** Table of reported cases of NUT carcinoma found outside of the midline region in the literature.

Author	Year	Tumor primary site	Fusion	Patient age	Gender	Metastasis
Neumann et al.	2022	Thyroid	BRD4-NUT fusion	27 y.o.	Female	Yes
Kuo et al.	2021	Thyroid	BRD4-NUT fusion	34 y.o.	Male	No
Zhou et al.	2022	Thyroid	BRD4-NUT fusion	38 y.o.	Male	Not reported
Shehata et al.	2010	Pancreas	Not reported	2 y.o.	Male	Yes
Shehata et al.	2010	Supraorbital (left)	Not Reported	Newborn	Male	Yes
Mertens, et al.	2006	Iliac bone	BRD4-NUT fusion	10 y.o.	Male	No
Yang et al.	2021	Kidney	BRD4-NUT fusion	41 y.o.	Male	Yes
Sirohi et al.	2018	Kidney	BRD4-NUT fusion	36 y.o.	Female	Not reported
Ziai et al.	2010	Salivary gland (left submandibular)	BRD4-NUT fusion	15 y.o.	Male	No
den Bakker et al.	2009	Salivary gland (right parotid)	BRD4-NUT fusion	15 y.o.	Male	No
Bishop et al.	2016	Kidney	BRD4-NUT fusion	43 y.o.	Female	Not reported
Bishop et al.	2016	Sinonasal tract	Not reported	26 y.o.	Male	Not reported
Bishop et al.	2016	Sinonasal tract	Not reported	48 y.o.	Male	Not reported
Bishop et al.	2016	Lateral Neck	BRD4-NUT fusion	23 y.o.	Male	Not reported
Seim et al.	2022	Sublingual gland	BRD4-NUT fusion	26 y.o.	Male	Yes

Listed is the author, publication year, primary site, fusion, age of the patient, gender, and if the patient had/or developed metastases.

In our patient, the lesion was initially misdiagnosed as cholangiocarcinoma, and with Next-Generation RNA-based sequencing we were able to correctly diagnose the tumor. The diagnosis of NUT carcinoma can be accurately made with immunohistochemistry using a CLIA validated monoclonal antibody to NUT (87% sensitivity, 100% specificity) (21). However, this requires the pathologist and/or clinician to have a high clinical suspicion of the diagnosis. In this case, a young person with a cancer showing uncommon squamous differentiation should prompt consideration of NUT IHC testing. Alternatively, wider access and availability of broad panels to include upfront RNA-based next-generation sequencing will lead to a diagnosis of this -fusion driven cancer. Our patient was treated with standard dose of Pembrolizumab alongside standard dose FOLFOX chemotherapy. A prior case report showed efficacy of 10 months survival on Pembrolizumab monotherapy after initial resection of the tumor; however, that patient also succumbed to their malignancy due to the aggressive nature of this cancer (13). Our patient survived well over a year and a half (13 months from time of diagnosis and surgery), one of the longest living NC of non-midline region origin. While on chemotherapy, she maintained a good quality of life and was able to perform her daily living activities without much sufferance.

The future of NC research appears promising. Although frequently misdiagnosed, proper IHC panel testing with the surge of next-generation sequencing may properly identify many more cases at an earlier time. Oral bromodomain and extra-terminal domain inhibitors (BET-inhibitors) have been used and have shown initial promise in NUT carcinoma, with combination clinical trials underway eg NCT05019716 (1, 8, 21).

## Data availability statement

The raw data supporting the conclusions of this article will be made available by the authors, without undue reservation.

## Ethics statement

The studies were conducted in accordance with the local legislation and institutional requirements. The participants provided their written informed consent to participate in this study. Written informed consent was obtained from the individual(s) for the publication of any potentially identifiable images or data included in this article.

## Author contributions

BA: Writing – original draft, Writing – review & editing. JA: Writing – original draft, Writing – review & editing. JL: Writing – original draft, Writing – review & editing. CF: Writing – review & editing.
